# How the Leopard Hides Its Spots: *ASIP* Mutations and Melanism in Wild Cats

**DOI:** 10.1371/journal.pone.0050386

**Published:** 2012-12-12

**Authors:** Alexsandra Schneider, Victor A. David, Warren E. Johnson, Stephen J. O'Brien, Gregory S. Barsh, Marilyn Menotti-Raymond, Eduardo Eizirik

**Affiliations:** 1 Laboratório de Biologia Genômica e Molecular, Faculdade de Biociências, Pontifícia Universidade Católica do Rio Grande do Sul (PUCRS), Porto Alegre, Brazil; 2 Laboratory of Genomic Diversity, Frederick National Laboratory for Cancer Research, Frederick, Maryland, United States of America; 3 Theodosius Dobzhansky Center for Genome Informatics, St. Petersburg State University, St. Petersburg, Russian Federation; 4 HudsonAlpha Institute for Biotechnology, Huntsville, Alabama, United States of America; 5 Instituto Pró-Carnívoros, Atibaia, São Paulo, Brazil; Texas A&M University, United States of America

## Abstract

The occurrence of melanism (darkening of the background coloration) is documented in 13 felid species, in some cases reaching high frequencies at the population level. Recent analyses have indicated that it arose multiple times in the Felidae, with three different species exhibiting unique mutations associated with this trait. The causative mutations in the remaining species have so far not been identified, precluding a broader assessment of the evolutionary dynamics of melanism in the Felidae. Among these, the leopard (*Panthera pardus*) is a particularly important target for research, given the iconic status of the ‘black panther’ and the extremely high frequency of melanism observed in some Asian populations. Another felid species from the same region, the Asian golden cat (*Pardofelis temminckii*), also exhibits frequent records of melanism in some areas. We have sequenced the coding region of the *Agouti Signaling Protein* (*ASIP*) gene in multiple leopard and Asian golden cat individuals, and identified distinct mutations strongly associated with melanism in each of them. The single nucleotide polymorphism (SNP) detected among the *P. pardus* individuals was caused by a nonsense mutation predicted to completely ablate ASIP function. A different SNP was identified in *P. temminckii*, causing a predicted amino acid change that should also induce loss of function. Our results reveal two additional cases of species-specific mutations implicated in melanism in the Felidae, and indicate that *ASIP* mutations may play an important role in naturally-occurring coloration polymorphism.

## Introduction

Melanism is a remarkable polymorphic phenotype observed in multiple animal groups, whose occurrence may be influenced by differential adaptation to varying environments or to distinct inter-specific interactions [1]–[3]. In the cat family (Felidae), melanism is quite common, having been reported in 13 of 37 extant species (Table 1). Although such darkened pelage reaches considerably high frequencies in some cat species [4], supporting the notion that this phenotype may be adaptive in some contexts, still little is known about its evolutionary history and ecological/behavioral significance in any felid. Initial molecular analyses have revealed that melanism arose multiple times in the Felidae, with three different mutations being implicated in this phenotype in distinct species [5].

**Table 1:** Available information on the occurrence of melanism in felid species.

Species	Strongest evidence and original references	Proposed mode of Inheritance	No. of offspring analyzed in the original literature source
***Felis catus***	**Visual** [30], [31]	**Recessive** [5], [30], [31]	**1 black offspring from a pair of wild type parents** [30], [31]
***Felis chaus***	**Photograph** [32]	**Dominant** [32]	**1 wild-type offspring from a pair of melanistic parents** [32]
*Felis silvestris, F. lybica*	Anecdotal [32], [33]	-	-
*Prionailurus bengalensis*	Anecdotal [34], [35]	-	-
***Panthera pardus***	**Visual** [36], [37]	**Recessive** [36], [37]	**Total of 439 offspring** [36], [37]
***Panthera onca***	**Visual** [32]	**Dominant** [5], [32]	**Total of 81 offspring** [32]
*Panthera leo*	Anecdotal [32]	-	-
*Panthera tigris*	Anecdotal [34], [38]	-	-
*Panthera uncia*	Anecdotal [39]	-	-
*Neofelis nebulosa*	Anecdotal [40], [41]	-	-
***Lynx rufus***	**Photograph** [34]	-	-
***Leopardus geoffroyi***	**Visual** [42]	-	-
***Leopardus guigna***	**Photograph** [32], [43], [44], [45]	-	-
***Leopardus tigrinus***	**Visual** [32], [46]	-	-
***Leopardus colocolo***	**Photograph** [32]	**Recessive** [32]	**2 black offspring from a pair of wild-type parents** [32]
			
*Acinonyx jubatus*	Anecdotal [40], [47] *	-	-
*Puma concolor*	Anecdotal [48]	-	-
***Puma yagouaroundi***	**Visual** [5]	**Co-dominant** [5]	-
***Leptailurus serval***	**Video** [33], [34], [39]	**-**	**-**
*Caracal caracal*	Anecdotal [34]	-	-
*Caracal aurata*	Anecdotal [49]	-	-
***Pardofelis temminckii***	**Photograph** [34], [35]	**Recessive** **	**-**
***Pardofelis marmorata***	**Photograph** [50]	**-**	**-**

Bold types indicate species for which reliable evidence of melanism exists (including direct visual observation by E.E., photograph, or video). Numbers refer to bibliographic sources (see References).

* Reference to melanism is not explicit.

** Based on results from this study.

As is the case in other vertebrates [1], [6], felid melanism was found to be influenced by two different genes whose products interact in the regulation of melanin production. Eumelanin (dark pigment) is produced when the Melanocortin-1 receptor (MC1R) is activated by the binding of Alpha Melanocyte Stimulating Hormone (α-MSH). In contrast, MC1R activation is inhibited by the binding of the antagonist peptide ASIP (Agouti Signaling Protein), whose action leads to a switch to pheomelanin (light pigment) synthesis [2], [7], [8]. Therefore, gain of function in MC1R or loss of function in ASIP induce melanism. In felids, both genes were found to be implicated, with *MC1R* variants underlying melanistic phenotypes in two different wild cat species (*Panthera onca* and *Puma yagouaroundi*), and a mutation in *ASIP* inducing black color in domestic cats [5].

Since that initial study, no additional mutation involved in melanism has been identified in any of the remaining felid species exhibiting this trait, hampering a broader assessment of its evolutionary history and adaptive significance. Such lack of knowledge is remarkable, as it extends to well-known and iconic animals such as the ‘black panther’, the melanistic form of the leopard (*Panthera pardus*) that is very common in some regions of southeastern Asia and often seen in zoos and museums. Other wild cats exhibiting melanism are less known, and the molecular analysis of melanism-inducing mutations would provide relevant insights into even basic aspects of the biology of this polymorphic phenotype in the wild.

In this study we report two novel mutations associated with melanism in wild felids, demonstrating that this mutant phenotype arose at least five times independently in the cat family. We show that two different variants of the *ASIP* gene are implicated in melanistic phenotypes in the leopard and in the Asian golden cat (*Pardofelis temminckii*). We discuss these findings in the context of the evolution of melanism, as well as the relative roles of *ASIP* and *MC1R* in the origin of such pigmentation variants.

## Materials and Methods

### Ethics statement

Biological samples used in this study were available in the tissue collection held at the Laboratory of Genomic Diversity, National Cancer Institute, National Institutes of Health (USA), having been collected previously in the context of collaborations with the South East Asian Zoological Park and Aquarium Association (SEAZA), the Chinese Association of Zoological Gardens (CAZG) and multiple captive breeding institutions from several countries (listed on Table 2). The purpose of those collaborations was to collect biological materials from a representative sample of Southeast Asian wild felids to allow studies on their taxonomy, genetics, evolution, and epidemiology, whose results would be incorporated into the design and implementation of conservation strategies on behalf of these species. Samples were collected by trained and certified veterinarians in the course of general health check-ups, following protocols approved by the scientific and/or ethics committees of each captive breeding institution. After collection, samples were imported into the USA under CITES permit number 12US694126/9, issued to the Laboratory of Genomic Diversity, National Institutes of Health, USA.

**Table 2:** Samples of *Panthera pardus* and *Pardofelis temminckii* included in the present study, including their respective genotypes for *ASIP*.

Sample ID^a^	Origin	Institution/Contact	Coat Color	*ASIP*	
				Genotype	positions
				333	384
**Ppa-221**	Jenderak, Malaysia	Melaka Zoo, Malaysia	Melanistic	A/A	C/C
**Ppa-222**	Negeri Sambilay, Malaysia	Melaka Zoo, Malaysia	Melanistic	A/A	C/C
**Ppa-223**	Perak, Malaysia	Melaka Zoo, Malaysia	Melanistic	A/A	C/C
**Ppa-224**	Jenderak, Malaysia	Melaka Zoo, Malaysia	Melanistic	A/A	C/C
**Ppa-225**	Dungun, Malaysia	Melaka Zoo, Malaysia	Melanistic	A/A	C/C
**Ppa-227**	Taiping, Malaysia	Taiping Zoo/Kevin Lazarus	Melanistic	A/A	C/C
**Ppa-228**	Taiping, Malaysia	Taiping Zoo/Kevin Lazarus	Melanistic	A/A	C/C
**Ppa-230**	Pehang Pekan, Malaysia	Negara Zoo	Melanistic	A/A	C/C
**Ppa-231**	Johor, Malaysia	Negara Zoo	Melanistic	A/A	C/C
**Ppa-284**	Guamurang, Malaysia	Khao Kheow Open Zoo	Melanistic	A/A	C/C
**Ppa-288**	Chiangmai Zoo, Thailand	Warren Johnson	Melanistic	A/A	C/C
Ppa-277	Probably Thailand	Khao Kheow Open Zoo	Wild-type	C/A	C/C
Ppa-283	Probably Thailand	Khao Kheow Open Zoo	Wild-type	C/C	C/C
Ppa-285	Chonburi, Thailand	Khao Kheow Open Zoo	Wild-type	C/C	C/C
Ppa-286	Chonburi, Thailand	Khao Kheow Open Zoo	Wild-type	C/C	C/C
**Pte-038**	Bangkok, Thailand	Dusit Zoo	Melanistic	C/C	G/G
**Pte-051** b	Yunnan, Ruili Region, China	Kunming Zoo	Melanistic	C/C	G/G
Pte-052^b^	Gansu Province, Tianshui Region, China	Lanzhou Zoo	Wild-type	C/C	C/C
Pte-053^b^	Gansu Province, Tianshui Region, China	Lanzhou Zoo	Wild-type	C/C	C/C

Melanistic individuals are highlighted in bold.

a Code names indicate species identification of each sample: Ppa = *Panthera pardus*; Pte = *Pardofelis temminckii*.

b Individuals shown in Figure 2: Pte-051 in panel E, Pte-052 in panel D and Pte-053 in panel C.

### Methods

The study was performed on the basis of biological material (blood or skin samples) of *P. pardus* and *P. temminckii* collected from captive animals of Asian origin (Table 2). In order to minimize any impact of population structure on the association studies, we strived to only include samples that were originated from the same geographic region or nearby locations for each of the species.

DNA extraction from all samples was performed using standard phenol/chloroform protocols [9]–[11]. To identify potential molecular variants associated with melanistic coat color in these species we characterized the candidate gene *ASIP*. The coding region of the gene was amplified by PCR (*Polymerase Chain Reaction*; [12]) from each sample, using primers designed with the software Primer3 (http://frodo.wi.mit.edu; see Table S2 for primer sequences) [13] on the basis of the domestic cat genomic sequence (U. *California - Santa Cruz*, http://genome.ucsc.edu/; *GARFIELD*, http://lgd.abcc.ncifcrf.gov/cgi-bin/gbrowse/cat/). PCR reactions for *ASIP* exon 2 and exon 3 were performed in a 10 µL final volume containing 2.0 mM MgCl_2_, 0.2 mM dNTPs, 0.5 U of AmpliTaq Gold DNA polymerase (Applied Biosystems), 0.2 µM each of the forward and reverse primers, and 10 ng of DNA. Thermal cycling used a touchdown profile with the annealing temperature decreasing from 60°C to 51°C in 10 cycles, followed by 30 or 40 cycles with annealing at 50°C for exons 2 and 3, respectively. Amplification of *ASIP* exon 4 was carried out with Takara LA Taq with GC Buffer (Takara Bio Inc.), following the guidelines provided by the manufacturer and the same thermal cycling conditions as exon 3.

PCR products were purified with Exonuclease I and Shrimp Alkaline Phosphatase, and sequenced for both strands using BigDye chain terminator chemistry (Applied Biosystems). Sequencing products were purified using Sephadex G-50 plates and analyzed with an ABI 3700 automated DNA sequencer. All resulting sequences were analyzed with Sequencher 4.2 (GeneCodes Corporation, Ann Arbor, MI), and every polymorphism was carefully inspected for confirmation. Nucleotide and amino acid sequences of *ASIP* were aligned with multiple mammalian homologs using ClustalW (http://www.ebi.ac.uk/Tools/msa/clustalw2/), with alignments being subsequently inspected and verified by hand. The DNA sequences reported here have been deposited in GenBank (accession numbers JX845175-JX845178).

## Results and Discussion

### Identification of *ASIP* mutations

**Figure 1:**
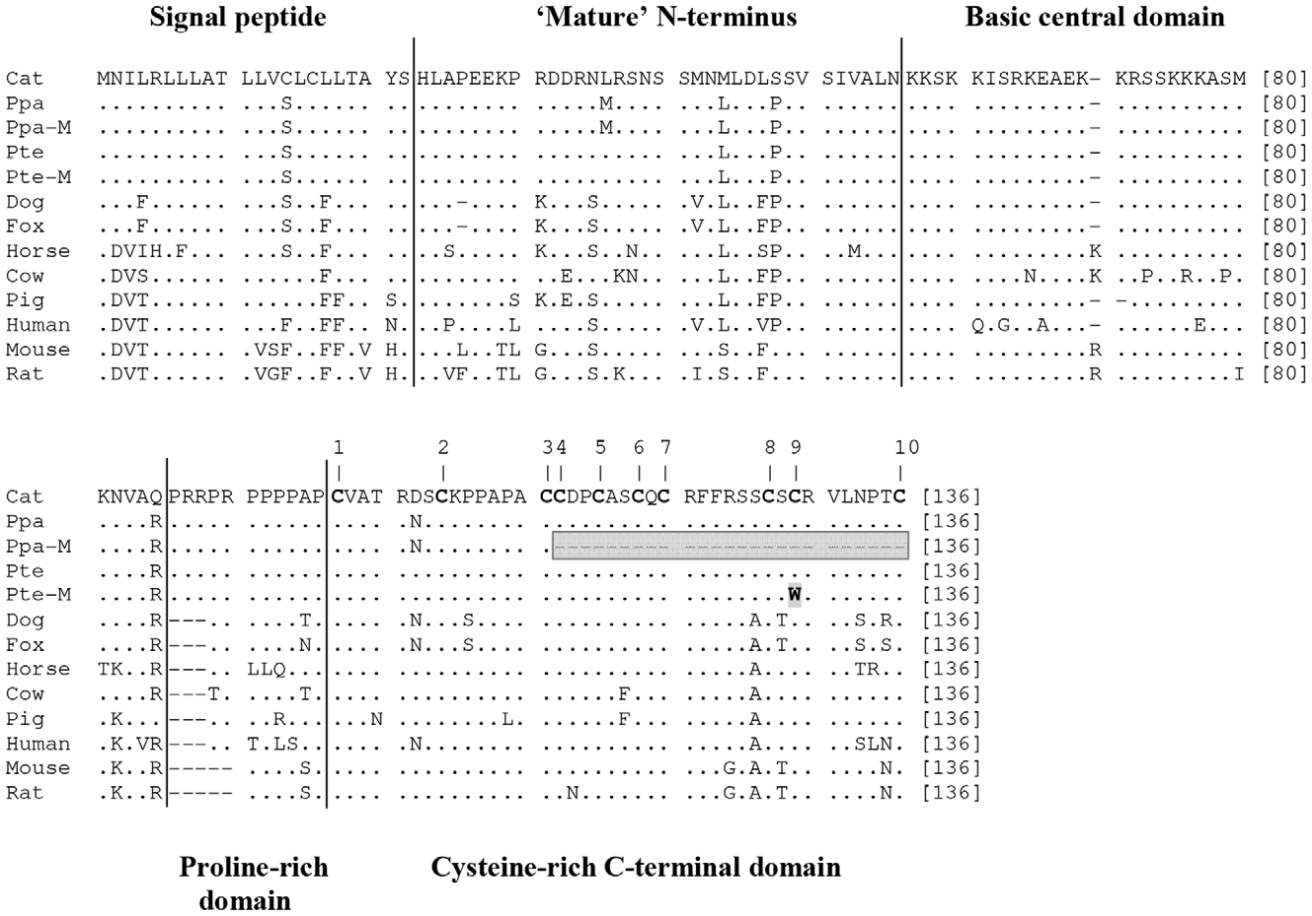
Amino acid alignment of ASIP, including the novel *Panthera pardus* and *Pardofelis temminckii* sequences. Wild-type and melanistic sequences of each wild cat species are shown. Dots indicate identity to the top sequence; amino acid positions are shown at the end of each line. Vertical lines demarcate the boundaries among the five functional domains proposed for ASIP ([17]), named above or below the sequences. Dashes represent insertion/deletion (indel) variants. Numbers 1–10 refer to the 10 conserved cysteine residues present in the C-terminal domain. The premature stop codon in melanistic *P. pardus* is shaded (dashes indicate deleted sites). The non-synonymous mutation in melanistic *P. temminckii* is indicated in bold and shaded as well.

**Figure 2:**
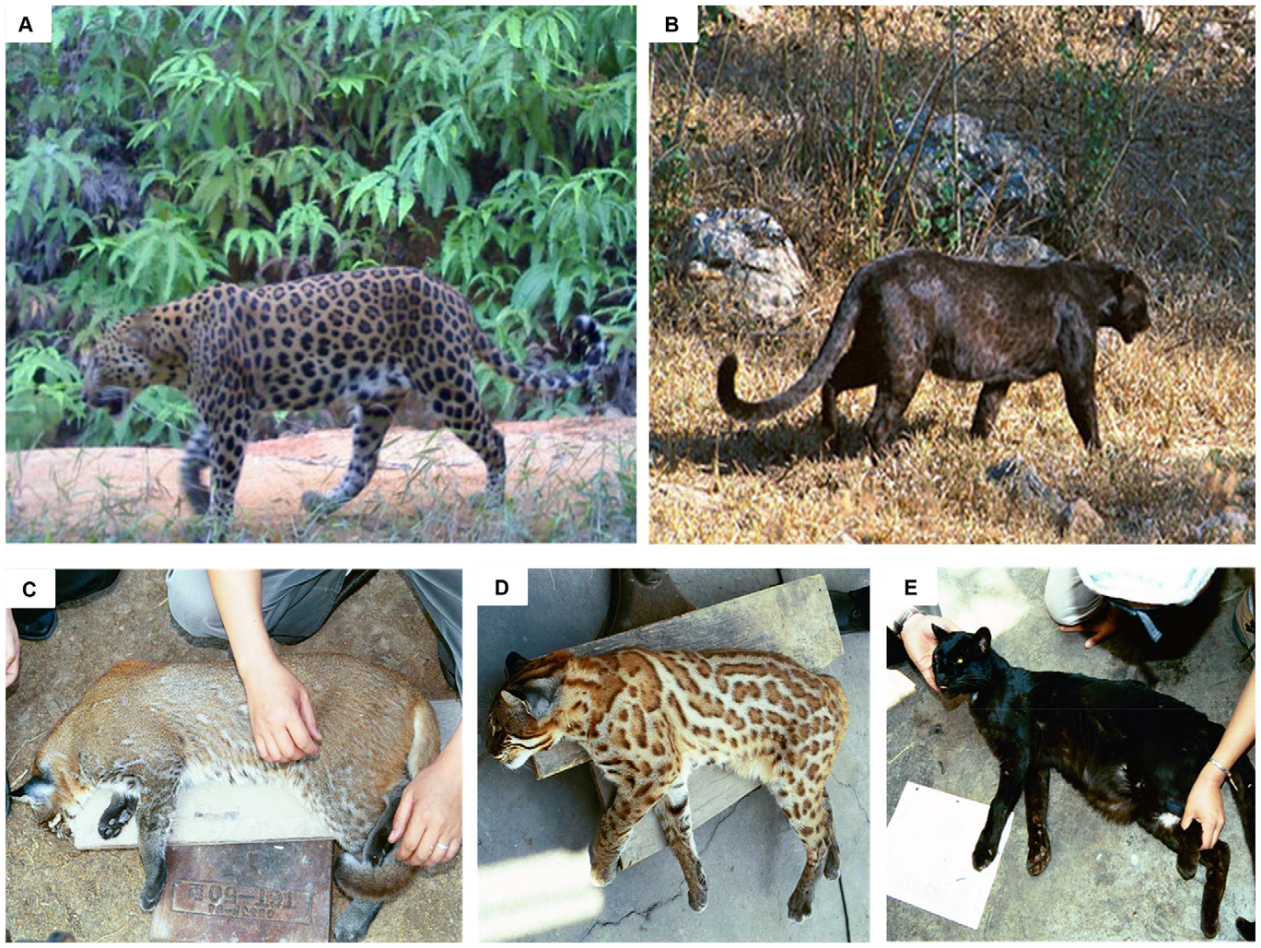
Coat color phenotypes of the leopard (*Panthera pardus*) (top) and Asian golden cat (*Pardofelis temminckii*) (bottom). (A) Typical non-melanistic leopard individual. (B) Melanistic leopard or ‘black panther’. (C, D, E) Polymorphic coat color of *P. temminckii*: (C) plain agouti with few markings; (D) tan background with dark rosettes; (E) melanistic phenotype. The individuals shown in C, D and E were actually typed in this study (see Table 2). Photo credits to Kae Kawanishi (A), Bruce Kekule (B), Warren Johnson and Sujin Luo (C, D, E).

Sequencing of the coding region of *ASIP* revealed that it was highly conserved within each species, with all individuals exhibiting an identical sequence except for a single nucleotide site (Figures 1 and S1). The single nucleotide polymorphism (SNP) detected among the *P. pardus* individuals was caused by a non-synonymous mutation located in exon 4 (C333A) predicted to introduce a stop codon at amino acid position 111. All 11 analyzed melanistic leopards (Figure 2) were homozygous for this mutation, while the wild-type individuals (i.e. bearing a yellowish background coloration with black rosettes; see Figure 2) were either homozygous for the ancestral ‘A’ allele or heterozygous. This finding reveals a significant association between melanism and a homozygous AA genotype (χ^2^ = 14.95, d.f. = 1, p<0.005), which is consistent with a recessive mode of inheritance of this trait in leopards, as suggested by previous breeding studies performed in captivity (Table 1).

A different SNP was identified in exon 4 of *P. temminckii*. The ancestral allele was identified by comparison to sequences from other species, and consists of a ‘C’ at position 384 (see Figure S1). The mutant allele derives from a non-synonymous substitution (C384G) predicted to cause a cysteine-tryptophan substitution at codon 128 (see Figure 1). This mutant allele was perfectly associated with black coat color in the Asian golden cat (χ^2^ = 4.00, d.f. = 1, p<0.05). The melanistic individuals (n = 2; see Figure 2E) were homozygous for the mutant allele, whereas two non-melanistic animals (one of which was plain agouti-colored and the other bearing dark rosettes; see Figure 2C, 2D) were homozygous for the ancestral allele. Given that *ASIP*-associated melanism is always inherited as a recessive trait [14], [15] we can infer that this is the mode of inheritance in Asian golden cats, as observed in leopards and also in domestic cats (see Table 2). As *P. temminckii* has been the focus of very few genetic studies, so far the inheritance mode of this prominent coloration polymorphism had remained unknown for this species.

### Comparative analysis of *ASIP* variation

We aligned our *ASIP* coding sequences to those generated previously for other mammals (see Table S1). The alignment consisted of 408 bp (136 codons) that exhibited heterogeneous patterns of variation. Some sites were highly conserved across mammals, whereas other segments were quite variable at the nucleotide and amino acid levels (see Figure 1 and Figure S1). A highly variable region, including multiple substitutions as well as insertion/deletion (indel) sites, was located between nucleotide coding positions 240 and 290, at the boundary between the basic (lysine-rich) and proline-rich central domains. At the amino acid level, this region was also considerably variable, but even higher diversity was observed in portions of the signal peptide and the mature N-terminus. Such variation may be due to relaxation of functional constraints in these regions, or to diverging selective pressures across lineages. Testing these hypotheses would help understand the historical pressures shaping *ASIP* diversity in mammals, and could be accomplished with structural and molecular evolutionary analyses targeting these particular regions of the gene.

In contrast to these highly variable segments, some regions were quite conserved across mammals, including sites that have remained identical in all the species sampled so far (Figure 1 and Figure S1). Some conserved amino acid sites are particularly noteworthy, as they have been the subject of direct experimentation assessing their functional relevance [16], [17]. All the amino acid residues in which replacements have been experimentally shown to cause loss or decrease of *ASIP* function are completely conserved across mammals. In particular, these experiments revealed that non-synonymous mutations involving each of the 10 cysteine residues of the C-terminal Cys-rich domain negatively affected ASIP activity. Eight out of 10 substitutions (at cysteine sites 1–4 and 6–9 (see Figure 1)) abolished ASIP activity, while two others (at sites 5 and 10) resulted in partial loss of protein function. Therefore, these cysteine residues were found to be critical for protein activity and receptor binding [16]–[19].

Such direct experimental evidence facilitates the interpretation of novel mutations affecting some of these conserved residues. The amino acid change associated with melanism in *P. temminckii* affects the 9^th^ conserved cysteine residue (see Figure 1), which was shown in mice to be required for ASIP function, and whose loss led to melanism [16]. Even stronger impacts are expected from mutations that induce stop codons in this region, as they can remove more than a single conserved cysteine residue. In mice, a mutation affecting the 5^th^ cysteine introduced a stop codon that led to a null phenotype [17], while mutations inducing premature stop codons (also removing conserved cysteines) in other species were associated with melanistic phenotypes as well [5], [20]. In this context, the mutation identified in black leopards is inferred to have a substantial functional impact, eliminating most of the C-terminal domain, from the 4^th^ conserved cysteine onward. Overall, these observations reinforce our inference that both mutations detected in wild cats are likely to cause melanism due to loss of ASIP function.

### Melanism Evolution in the Felidae

Although it is often difficult to demonstrate a clear association between coat color polymorphism and SNP variation [21], [22], there have been several examples of success in identifying mutations implicated in melanism. In almost every case they were variants of the *ASIP* or *MC1R* genes, which were associated with darkened phenotypes in domestic and wild populations [6], [23]–[25]. In this context, a particular group that has been found to harbor species-specific mutations in these genes that are strongly associated with melanism is the family Felidae.

Our present results reveal two novel mutations implicated in melanism in felids. Taken together with the previous findings reporting three additional mutations [5], we conclude that this mutant phenotype arose at least five times independently in the cat family. Interestingly, three of these mutations are located in *ASIP*, indicating that this gene is equally or more often involved in felid melanism than *MC1R*.

This observation contrasts with the view that *MC1R* is more frequently implicated in melanism than *ASIP* [1], [8]. Kingsley et al. [20] have hypothesized that the perceived higher frequency of *MC1R*-induced melanism in natural populations, relative to *ASIP*-induced darkening, may be due to either lower pleiotropic effect of mutations in the former, or to differential effects of natural selection on variants of each gene. Given current knowledge on their biology, it is unclear whether *ASIP* mutations would have substantially more pleiotropic effects than those in *MC1R*. In effect, the *ASIP* coding region is quite variable across taxa (see Figure 1), suggesting that functional constraints on this gene are not very stringent. Additional functional studies are thus required to assess in more detail the pleiotropic effects of both loci. In addition, it remains possible that, due to lineage-specific genetic features, *ASIP* mutations are less affected by pleiotropic effects in felids, allowing this gene to be less constrained and thus more often involved in melanistic phenotypes. This hypothesis can be tested by investigating differential patterns of expression and activity of *ASIP* in felids relative to other groups.

Another interesting aspect pertains to the relevance of regulatory vs. coding mutations in the context of *ASIP*-induced melanism. Although it has been proposed that *ASIP*-related melanism is more often caused by regulatory mutations [8], [21], our results show a high incidence of coding mutations leading to pelage darkening in felids. Again, this may be a consequence of felid-specific changes in the pleiotropic effect of *ASIP* mutations, which is likely stronger when the coding region is affected [20]. Remarkably, the three different *ASIP* mutations found so far to induce melanism in felids seem to cause complete loss of gene function, and might therefore induce strong pleiotropic effects. Nevertheless, there is so far no evidence of pleiotropic effects associated with melanism in domestic or wild felids, suggesting that loss of ASIP function only affects pigmentation, or can be compensated in other systems by the activity of other proteins.

The second hypothesis raised by Kingsley et al. [20] to explain the apparent difference in *ASIP vs. MC1R* involvement in melanism pertains to differential effects of natural selection on these loci. Since melanism is dominant when induced by *MC1R*, it is more easily detected by natural selection, and would more quickly rise in frequency when favorable. On the other hand, *ASIP*-induced melanism is recessive, and would thus take more time to rise in frequency when favorable, but also linger in the population for a longer period when negatively selected. Kingsley et al. [20] thus hypothesized that *MC1R*-induced melanism would be prevalent when this trait is adaptive, but *ASIP*-induced darkening might be expected when the trait is deleterious. This would more often occur when melanism is present at low frequencies, as was the case in the *Peromyscus* populations analyzed by Kingsley et al. [20]. In contrast, *ASIP*-induced melanism can reach very high frequency in some felid populations, suggesting that this trait may be adaptive or at least neutral.

Such a pattern is particularly noticeable in the case of leopards from the Malay Peninsula, where melanism approaches fixation [4]. Using samples from this very region (see Table 2), we show here that *ASIP* is implicated in this mutant phenotype. Although we have shown that this near fixation may have been caused by genetic drift over a long period of time [4], this would be very unlikely if the trait was deleterious. Moreover, such high frequency would be much more quickly achieved if the trait was favorable, and therefore driven to near fixation by natural selection. The identification of the molecular basis of this phenotype now opens up new avenues to investigate its evolutionary history and adaptive significance in the wild.

Another interesting point regarding leopard melanism is the observation that black rosettes are still visible in spite of the much darkened background coloration (see Figure 2B). This indicates that rosettes are still darker than the essentially black background, and are not obliterated by the melanism-inducing mutation. Such observation supports the hypothesis that pattern formation on mammalian coats is induced by two separate processes, encompassing considerably more complexity than the well-established ASIP-MC1R interplay [26]–[28]. Although it could be hypothesized that localized differences in ASIP and/or MC1R expression/function could induce the presence of spots/stripes on mammalian coats, observations such as the presence of these ‘ghost rosettes’ argue otherwise. Moreover, the results from this study indicate that melanism in leopards is caused by complete loss of ASIP function, which would imply no action of this antagonist peptide and thus maximum MC1R signaling for dark melanin across the whole body. The fact that rosettes are even darker than this background strongly argue for the action of a distinct pigmentation pathway [28], which has so far not been characterized in any mammal bearing ASIP-null mutations [7], [25], [29]. Interestingly, in black domestic cats (also inferred to be induced by loss of ASIP function [5]), ‘ghost’ tabby markings are mostly visible in the juvenile, and become indistinguishable from the darkened background in the adult. Dissecting the molecular and developmental pathways affecting coat patterning *vs.* background melanogenesis in these and other felid species promises to shed unprecedented light onto the genetic basis and evolutionary history of pigmentation diversity in mammals.

## Supporting Information

Figure S1 **Nucleotide variation in the** ***ASIP*** **coding region among mammals, including sequences of** ***Panthera pardus*** **and** ***Pardofelis temminckii*** **, shown for a wild-type and a melanistic individual (indicated by the letter ‘M’).** Asterisks indicate the nucleotide position for the mutant alleles associated with melanism. Dots indicate identity to the top sequence; vertical lines demarcate boundaries between exons. Shaded segments containing dashes indicate insertion/deletion (indel) regions.
(DOC)

Table S1 **GenBank accession numbers for mammalian sequences included in the** ***ASIP*** **alignments analyzed in this study.**
(DOC)

Table S2 **Primers developed in this study for PCR amplification and sequencing of** ***ASIP*** **in felids.**
(DOC)
