# Supplementary material for: How the Leopard Hides Its Spots: ASIP Mutations and Melanism in Wild Cats
Source: PLoS One. 2012 Dec 12;7(12):e50386. doi: 10.1371/journal.pone.0050386 (PMC3520955; doi:10.1371/journal.pone.0050386)
Supplement: Figure S1 — Nucleotide variation in the ASIP coding region among mammals, including sequences of Panthera pardus and Pardofelis temminckii , shown for a wild-type and a melanistic individual (indicated by the letter ‘M’). Asterisks indicate the nucleotide position for the mutant alleles associated with melanism. Dots indicate identity to the top sequence; vertical lines demarcate boundaries between exons. Shaded segments containing dashes indicate insertion/deletion (indel) regions. (DOC) [file pone.0050386.s001.doc]

Figure S1

Cat ATGAATATCC TCCGCCTACT CCTGGCCACC CTGCTGGTCT GCCTGTGCCT CCTCACTGCC TACAGTCACC TGGCACCTGA GGAAAAACCC AGAGATGACA [100]

Ppa .......... .......... .......... .......... C......... .......... .......... .......... .......... .......... [100]

Ppa-M .......... .......... .......... .......... C......... .......... .......... .......... .......... .......... [100]

Pte .......... .......... .......... .......... C......... .......... .......... .......... .......... .......... [100]

Pte-M .......... .......... .......... .......... C......... .......... .......... .......... .......... .......... [100]

Dog .........T .......... .......... .......... C.......T. .......... .....C.... ....T---.. ......G... .AG....... [100]

Fox .........T .......... .......... .......... C.......T. .......... .....C.... ....T---.. ......G... .AG....... [100]

Horse ...G..G..A .T.A...GT. .......... .........A ....C...T. .......... .....C.... ..T....... ...G..G... .A........ [100]

Cow ...G..G..A G......C.. ......T... T......... ........T. .......... .....C.... .......... ......G... ........A. [100]

Pig ...G..G..A CT.....C.. .T.A..T... ..A.....A. ........T. .T........ .C...C.... .......... ......GT.. .A......A. [100]

Human ...G..G..A C....T.... .......... .......... T...C...T. .T........ A....C.... ..C....... ...G..G.T. C......... [100]

Mouse ...G..G..A C......... .......... ..AG..AG.. T.......T. .T....C.T. C....C.... ......TC.. ...G.CG.TT G......... [100]

Rat ...G..G..A C......... .......... ..CG...G.. T.......T. ......C.T. C....C.... ...T.TT... ...G.CG.TT G......... [100]

Cat GGAACCTGAG GAGCAACTCC TCCATGAACA TGTTGGATCT CTCTTCTGTC TCTATTGTAG CGCTGAACAA GAAATCCAAA AAGATCAGCA GAAAAGAGGC [200]

Ppa .....A.... .......... .........C .......... .C........ .......... .A........ .......... .......... .......... [200]

Ppa-M .....A.... .......... .........C .......... .C........ .......... .A........ .......... .......... .......... [200]

Pte .......... .......... .........C .......... .C........ .......... .A........ .......... .......... ....G..... [200]

Pte-M .......... .......... .........C .......... .C........ .......... .A........ .......... .......... ....G..... [200]

Dog ...G...A.. .......... ..TG.....C .T......T. .C........ .......... .A........ .......... .......... .......... [200]

Fox ...G...A.. .......... ..TG.....C .T......T. .C........ .......... .A........ .......... .......... .......... [200]

Horse ...G...... ..A....... .........C ........TC .C........ .....CA.G. .AT....... .......... .......... .......A.. [200]

Cow .........A ..A......T .........C ........T. .C....A... .....C..G. .......... .......... .......... ....T..A.. [200]

Pig .A.GT..A.. .......... .........C ........T. .C........ ........G. .A........ .......... .......... .......A.. [200]

Human ...G...... A......... ..TG.....C .AC.....G. .C........ ........G. .......... .......... C.....G... ......CA.. [200]

Mouse ...GT...C. ...T...... .........T C.C.....T. ...C.....T .....C..G. .A........ .........G .......... .......A.. [200]

Rat ...GT..A.A .........T .....C...T CAC.....T. ...C.....T ..C.....G. .A........ .........G .......... .......A.. [200]

Cat GGAAAAG--- AAGAGATCTT CCAAG AAAAA GGCTTCGATG AAGAATGTTG CTCAGCCTCG GCGGCCCCGG CCTCCGCCGC CCGCCCCCTG CGTGGCCACT [300]

Ppa A......--- .......... ..... ..... .......... .......... ...G...... .......... .......... .......... .......... [300]

Ppa-M A......--- .......... ..... ..... .......... ......... ...G...... .......... .......... .......... .......... [300]

Pte .......--- .......... ..... ..... .......... .....C.... ...G...... .......... .......... .......... .......... [300]

Pte-M .......--- .......... .......... .......... ......C.... ...G...... .......... .......... .......... .......... [300]

Dog .......--- .......... .T... ..... .......... .....C..G. ...GT----- ----...... ..C.....A. ..A....... .......... [300]

Fox .......--- .......... .T... ..... .......... .....C..G. ...GT----- ----...... ..C.....A. ..AA...... .......... [300]

Horse A......AAG .......... ..... ..... .......... .C...G..G. .G.G.----- ----.....T .TC.T..A.. .......... T........C [300]

Cow .......AAG ..A...C... ..... .G... ....C..... .....C..G. .A.G.----- ----A..... ..C....... .TA....... .........C [300]

Pig ...G...--- ---....... ..... ..... .......... .....G..G. .A...----- ----...... ..C....G.. .T........ ........AC [300]

Human A......--- ..A....... .T... ..GG. .......... .....A..G. TG.G.----- ----...... A.C..C.TAT .T..G..... .........C [300]

Mouse C..G...CGG .....G.... ..... ..... .......... .....G..G. .AAG.----- ---------- ..C.....A. .TT.G..... .........C [300]

Rat ...G...CGG .....G.... ..... ..... .........A .....G..G. .A.G.----- ---------- ..C.....A. .TT.G..... .........C [300]

* *

Cat CGTGACAGCT GCAAGCCGCC GGCGCCCGCC TGCTGCGACC CGTGCGCCTC CTGCCAGTGC CGCTTCTTCC GCAGCTCCTG CTCCTGCCGA GTGCTCAACC [400]

Ppa ...A...... .......... .......... .......... .......... .......... .......... .......... .......... .......... [400]

Ppa-M ...A...... .......... .......... .....**A**.... .......... .......... .......... .......... .......... .......... [400]

Pte ..A....... .......... .......... .......... .......... .......... .......... .......... .......... .......... [400]

Pte-M ..A....... .......... .......... .......... .......... .......... .......... .......... ......**G**... .......... [400]

Dog ..CA...... .....T.C.. .......... .....T.... .C........ .......... .......... .....G.... .A.......C ..T....GT. [400]

Fox ..CA...... .....T.C.. .......... .....T.... .C........ .......... .......... .....G.... .A.......C ..T....GT. [400]

Horse ..C....... .......... .......... .......... .......... .........T .......... .....G.... .........T .......CT. [400]

Cow ..C....... .......T.. A......... .......... .........T .......... .......... .....G.... .........C .......... [400]

Pig ..C....... .......T.. .....T.... .......... .........T .......... .......... ....TG.... .........C .......... [400]

Human ..CA...... .......... ...A...... .......... .......... .......... .......... .....G.... .........C .......G.. [400]

Mouse ..C....... .......A.. C..A...... .......... .......... .......... ..T......G .....G.... .A....T... ..A....... [400]

Rat ..C....... .......... T......... ......A... .......... .......... ..T......G .....G.... .A.T.....C ..A....... [400]

Cat CCACCTGCTGA [411]

Ppa ........... [411]

Ppa-M ........... [411]

Pte ........... [411]

Pte-M ........... [411]

Dog ...GA...... [411]

Fox ...G....... [411]

Horse G.......... [411]

Cow ........... [411]

Pig ........... [411]

Human T..A....... [411]

Mouse ...A....... [411]

Rat ...A....... [411]
