# Supplementary material for: How the Leopard Hides Its Spots: ASIP Mutations and Melanism in Wild Cats
Source: PLoS One. 2012 Dec 12;7(12):e50386. doi: 10.1371/journal.pone.0050386 (PMC3520955; doi:10.1371/journal.pone.0050386)
Supplement: Table S1 — GenBank accession numbers for mammalian sequences included in the ASIP alignments analyzed in this study. (DOC) [file pone.0050386.s002.doc]

**Table S1.** GenBank accession numbers for mammalian sequences included in the *ASIP* alignments analyzed in this study.

| **Mammal species** | **GenBank Acession Number** |
| --- | --- |
| Domestic cat (*Felis catus*) | NP_001009190.1 |
| Dog (*Canis familiaris*) | NP_001007264.1 |
| Red fox (*Vulpes vulpes*) | Y09877.2 |
| Horse (*Equus caballus*) | AF288358.1 |
| Cow (*Bos taurus*) | X99692.1 |
| Pig (*Sus scrofa*) | AJ427478.2 |
| Human (*Homo sapiens*) | NM_001672 |
| Mouse (*Mus musculus*) | NM_015770.3 |
| Rat (*Rattus rattus*) | NM_052979.1 |
