# Supplementary material for: How the Leopard Hides Its Spots: ASIP Mutations and Melanism in Wild Cats
Source: PLoS One. 2012 Dec 12;7(12):e50386. doi: 10.1371/journal.pone.0050386 (PMC3520955; doi:10.1371/journal.pone.0050386)
Supplement: Table S2 — Primers developed in this study for PCR amplification and sequencing of ASIP in felids. (DOC) [file pone.0050386.s003.doc]

**Table S2.** Primers developed in this study for PCR amplification and sequencing of *ASIP* in felids*.*

| **Primer** | **Sequence (5’ – 3’)** |
| --- | --- |
| ASIP exon2-F | TCTGTTCCACTCAGGCCTTC |
| ASIP exon2-R | GGGTCAAGCTGGGCTACTTA |
| ASIP-exon3-F | CTCTTCTCCCACACCCTGAG |
| ASIP-exon3-R | CACCCCCACAATGAAAACTC |
| ASIP-exon4-F | GAGCAGACCCCGCTTTTC |
| ASIP-exon4-R | GCCTTGGAGGTGGGTGAG |
